# Perceptions of Chinese higher-vocational nursing program directors on digital transformation: A qualitative study

**DOI:** 10.1371/journal.pone.0346261

**Published:** 2026-04-08

**Authors:** Kaixuan Duan, Fangqun Mao, Ruiming Li, Xiaofeng Jin, Hao Huang, Meng Wen

**Affiliations:** 1 Xinchang College, Zhejiang Industry Polytechnic College, Shaoxing, Zhejiang, China; 2 Academic Affairs Office, Taishan Vocational College of Nursing, Taishan, Shandong, China; 3 School of Nursing, Gansu Health Vocational College, Lanzhou, Gansu, China; UTM Skudai: Universiti Teknologi Malaysia, MALAYSIA

## Abstract

**Background:**

Currently, most research on digital transformation in higher vocational education conducts macro-level analyses, and few comprehensive studies specifically address higher vocational nursing education. Moreover, research is notably lacking on the perceptions of Chinese higher vocational nursing program directors, and the prevalent utilization of digital tools within this field remains underexplored.

**Objective:**

To understand the perceptions and experiences of higher vocational nursing program directors regarding the use of digital transformation tools in nursing education.

**Methods:**

This qualitative study employed semistructured interviews conducted with 14 program directors from higher vocational nursing programs. The interview data were transcribed, and Colaizzi’s seven-step method was used for a subject analysis.

**Results:**

The study employed Colaizzi’s seven-step framework for inductive thematic analysis, with coding conducted collaboratively until data saturation was achieved. Analysis revealed four principal themes: (1) Recognition of digital education by program directors, where enthusiasm for enhanced engagement and transcended geographical barriers coexisted with unspoken doubts about resource quality and the authenticity of student participation; (2) Perceived status and impact of digital infrastructure, characterized by significant hardware investments yet inconsistent resource quality and utilization—revealing a fundamental gap between technological abundance and meaningful pedagogical integration; (3) Systemic Tensions and Barriers, exposing profound contradictions between policy aspirations and classroom realities, generational divides in educational philosophies among teachers, and persistent disconnects between technology’s promise and its operational friction, all of which contributed to increased workloads and a pervasive sense of professional role dissonance; (4) Strategic Approaches to Digital Transformation, centered on increasing investment, fostering educator development, promoting resource sharing, and strengthening industry-education integration.

**Conclusion:**

Higher vocational nursing program directors recognize the value of digital education, yet its adoption is constrained by challenges in platform usability and a lack of supportive institutional incentives. Effective transformation requires systemic strategies that enhance the digital ecosystem's ease of use, align professional rewards with innovation, and promote cross-sector collaboration to develop pedagogically sound resources.

## 1.  Background

With the rapid development of digital technologies, higher vocational nursing education is increasingly undergoing digital transformation. The UNESCO Statement on Ensuring Inclusive and Equitable Quality Education, along with the Promotion of Lifelong Learning Opportunities for All, is integral to The Global Education 2030 Agenda [1]. In the EU, the Digital Education Action Plan (2020) states that efficient and sustainable digital methods are part of a flexible education system. Furthermore, this plan encompasses strategies to improve digital competencies, promote digital literacy, and integrate digital technologies into educational practices [2]. In 2023, the 20^th^ National Congress of the Communist Party of China wrote “digital education” for the first time into a report [2] that established a plan for digital education as pivotal to the national development strategy and noted the direction for further improving the digital work of higher vocational education in the new era. These policy initiatives are supported by a growing body of empirical research examining the integration of digital technologies in health professions education [3–5],which has highlighted both the potential benefits and the implementation challenges faced by educators and institutions.All countries are actively promoting the digital transformation of education [4], including the digital transformation of nursing education. This global trend is reflected vividly in the distinct approaches of the United States and Europe. The U.S. leads the world in research output [5] and integrates frontier technologies such as mobile applications, gamification, and AI-generated content to foster interactive learning [3,6]. In contrast, Europe emphasizes structured digital competency frameworks and educator training [7] as well as the sustainable implementation of digital tools, as evidenced by multinational projects and post-pandemic evaluations [8,9]. These international experiences collectively highlight the critical role of technological innovation, pedagogical adaptation, and systemic support, providing a pertinent reference for digital transformation within China’s unique vocational education context.

Digital education encompasses teaching and learning activities facilitated by digital tools, the software and hardware that enable data processing in the modern era. Digital resources are curriculum education information presented through various means, such as videos, documents, animations, and virtual simulations [10]. Over the past half-century, digital education has emerged as a prevalent tool in health professional education. It offers a more flexible, accessible, and cost-effective alternative to conventional learning methods. Numerous scholarly articles have scrutinized the potential advantages of digital education in the context of health professional training [11,12]. For example, digitalization has facilitated active participation, learning, and collaboration among students, irrespective of temporal and location constraints [10]. Digital learning has facilitated the development of competence, thereby enhancing the understanding of skills relevant to health sciences [13]. Flexible, student-centered digital methods can bolster students’ metacognition, whereas digital learning has simultaneously increased student motivation, conserved resources, and expanded learning environments [14]. Digital platforms are already widely adopted, revolutionizing traditional teaching methods that rely on the demonstration and repetition of procedures [15]. Advancements in artificial intelligence technologies will further enhance learning analytics, thereby providing comprehensive support for both students and educators [16]. To a certain extent, these advancements require teachers to be proficient in using various digital tools and resources while addressing the challenges posed by the dynamic digital landscape.

At present, research on digital transformation in higher vocational education has predominantly focused on macro-level issues such as policy discussions, institutional strategies, and large-scale digital initiatives, whereas fewer studies have examined the practical experiences of program-level leaders responsible for implementing these initiatives on the ground. In particular, little is known about the practical use of digital tools by higher vocational nursing program directors and what challenges they encounter in this process.

The research questions were as follows:

1. How do Chinese higher vocational nursing program directors perceive the value and impact of digital transformation of education?
2. What challenges and difficulties do these program directors encounter during the implementation of digital transformation?
3. What strategies do these program directors propose to address these challenges and facilitate digital transformation?

## 2. Materials and methods

### 2.1.  Study design

This study adopted a qualitative methodology guided by an interpretive phenomenological approach, utilizing semi-structured interviews to explore the lived experiences and perceptions of Chinese higher vocational nursing program directors regarding educational digital transformation [18].

### 2.2. Participants

The study focused on nursing program directors. Although these individuals are experienced educators who are engaged in frontline teaching, they also hold key administrative roles. This dual perspective was purposively sought to gain insights into both the pedagogical realities and the strategic, resource-related decision-making processes that shape digital transformation at the institutional level. In this study, a purposeful sampling method was employed to recruit nursing program directors from the eastern, central, and western regions of China as participants.

The inclusion criteria were as follows: (1) program directors for nursing, directors of the teaching and research sections, academic leaders, deputy deans or department chairs of higher vocational nursing programs; (2) teaching experience of more than 3 years; (3) title of lecturer or chief nurse practitioner or higher; and (4) voluntary participation in the study with written informed consent. The exclusion criteria included (1) those who had not been involved in practical nursing teaching or leadership roles within the past year and (2) individuals who had never utilized any digital teaching methods in their actual teaching practices. The sample size was determined by data saturation, culminating in a final sample of 14 program directors from higher vocational nursing programs, labeled T1–T14. Among them, seven were from the eastern region of China, five were from the central region, and two were from the western region. Twelve participants were female, and two were male, with an average age of 40 years and an average teaching experience of 15 years. Detailed participant information is presented in Table 1.

**Table 1:** Participant characteristics(n = 14).

Participants	Gender	Age	Years’ experience	Educational background	Professional rank and title	Professional position	School
T1	Female	37	14	Master’s degree	Lecturer	Program director	Guangdong Z
T2	Female	46	22	Master’s degree	Associate professor	Vice Dean	Jiangsu Z
T3	Male	43	18	Bachelor’s degree	Associate professor	Department Head	Shandong T
T4	Female	40	13	Master’s degree	Associate professor	Program director	Fujian Q
T5	Female	39	13	Master’s degree	Associate professor	Program director	Fujian F
T6	Female	39	13	Master’s degree	Associate professor	Program director	Guangxi G
T7	Female	44	20	Bachelor’s degree	Lecturer	Program director	Guangxi W
T8	Female	39	14	Master’s degree	Lecturer	Program director	Hunan Y
T9	Female	39	13	Master’s degree	Associate professor	Vice Dean	Hunan X
T10	Female	40	12	Master’s degree	Lecturer	Program director	Hubei C
T11	Female	37	17	Master’s degree	Associate professor	Program director	Anhui W
T12	Female	49	25	Master’s degree	Associate professor	Program director	Heilongjiang H
T13	Male	30	5	Master’s degree	Lecturer	Program director	Chongqing C
T14	Female	34	11	Master’s degree	Lecturer	Program director	Gansu G

### 2.3.  Data collection

The data collection for this study was conducted by two researchers (Duan, Mao) from July 5th to August 3rd, 2024. Prior to the interviews, the participating program directors were informed of the research purpose, significance, and confidentiality principles and were provided written informed consent. One-on-one semistructured interviews were conducted in a quiet classroom at an agreed-upon time, with the entire session recorded upon the teachers’ consent. During the interviews, the researchers adjusted their questioning strategies on the basis of the teachers’ responses, observed their emotional changes, and made notes accordingly (Table 2). Each interview lasted from 30–60 minutes, and data saturation was achieved after the 14th participant was interviewed, as no new themes emerged from subsequent interviews, indicating that the collected data were comprehensive and representative of the target population [17].

**Table 2:** Interview questions.

No.	Questions
1	How do you view the role of digital transformation in empowering the development of nursing education and teaching in higher vocational education?
2	What is the current status of digital hardware construction in nursing at your school?
3	What is the current status of nursing digital software construction in your school?
4	What is the current status of nursing digital resource construction in your school?
5	How are digital software, hardware, and resources applied in various aspects of education and teaching? Please elaborate.
6	What factors do you think affect the development and application of digital nursing teaching in departments/faculties?
7	Regarding the factors you believe to be influential, how do you think they should be addressed?
8	How effective have been the teaching results achieved by applying digital teaching methods in your department? If they are effective, please share the possible reasons; if not, please also share the possible reasons.
9	How satisfied are you with the construction and application of digital education in your department/faculty?
10	Are you familiar with the current situation of digital transformation in national higher vocational nursing education? If so, please share your understanding of the current situation.

### 2.4.  Data analysis

The collected data were analyzed following Colaizzi’s phenomenological framework to explore the lived experiences of higher vocational nursing program directors [17].First, to become familiar with the data and assist in bracketing preconceptions, the interview recordings were transcribed verbatim and checked by author Duan. Duan and Mao then read and reread the transcripts independently to gain a sense of the whole response spectrum.This initial immersion was followed by a detailed analytic process. Second, significant statements pertaining to the phenomenon were identified. Following the principle of horizonalization, we extracted all statements relevant to the phenomenon, initially treating each statement as having equal analytic value before subsequent refinement.Third, the meanings embedded within these significant statements were formulated; this involved interpretive coding to capture the essence of each statement beyond its literal content. Fourth, through regular team meetings with discussions documented,the formulated meanings were discussed and clustered into themes that emerged from the data; disagreements were resolved through discussion until consensus was reached. The clustering step represented a fusion of horizons, linking individual experiences to the entire dataset. Fifth, exhaustive descriptions of the phenomenon were developed. Sixth, the fundamental structure of the experience was distilled. Finally, this essence was related back to the original transcripts for validation,and member checking was performed by returning a summary of the thematic structure to five participants for feedback; their feedback was used to refine phrasing and confirm that the interpretation aligned with their lived experiences.Throughout this iterative analytic process, we confirmed that thematic saturation had been reached during data collection, as no new thematic dimensions emerged from the ongoing analysis.

### 2.5.  Ethical consideration

Ethical approval for this study was obtained from the Dean of Xinchang College, Zhejiang Industry Polytechnic College, in accordance with the ethical principles of the Declaration of Helsinki, and the study protocol was further reviewed and approved by the Ethics Committee of Gansu Health Vocational College（No.2024020）. All participants were adult educators who provided written informed consent prior to data collection. To protect participant confidentiality, all of the data were anonymized, and any personally identifiable information was removed. The anonymized data were stored securely on password-protected university servers.

## 3.  Results

Following Colaizzi’s analytical framework, the interview data were examined rigorously. This process involved extracting significant statements from the transcripts, formulating their meanings, and clustering them iteratively into coherent categories. Through this inductive process, four major themes encapsulating the perceptions of Chinese higher vocational nursing program directors were derived: (1) the recognition of digital education by program directors; (2) the perceived status and impact of digital infrastructure; (3) the systemic tensions and barriers; and (4) strategic approaches to digital transformation.

The analysis yielded four principal themes that directly address the three research questions. Themes 3.1 and 3.2 correspond to the first research question regarding program directors’ perceptions of digital transformation. Theme 3.3 addresses the second research question concerning the tensions and barriers they encounter, while Theme 3.4 responds to the third research question by presenting the strategies they propose for facilitating digital transformation.The structure of these themes and their constituent subthemes, which illustrate the depth and variation in participant perspectives, is detailed in Table 3.

**Table 3:** Main themes and subthemes categorized from the study.

Main themes	Subthemes
3.1. Recognition of Digital Education by Program Directors	3.1.1. Teaching Enhancement
	3.1.2. Freedom and Loss of Control
	3.1.3. Student Engagement
3.2. Perceived Status and Impact of Digital Infrastructure	3.2.1. Resource Development
	3.2.2. Infrastructure Status
	3.2.3. Integrate Digital Resources into Lectures
	3.2.4. Application in Practical Teaching
3.3 Systemic Tensions and Barriers	3.3.1. Policy Practice Gap
	3.3.2. Readiness Divide
	3.3.3. Technical Operational Barriers
	3.3.4. Workload Pressures
	3.3.5. Professional Role Dissonance
3.4. Strategic Approaches to Digital Transformation	3.4.1. Infrastructure Investment
	3.4.2. Educator Development
	3.4.3. Resource Innovation
	3.4.4. Incentive Mechanisms
	3.4.5. Industry–Education Integration

### 3.1.  Recognition of digital education by program directors

#### 3.1.1.  Teaching enhancement.

Program directors were generally appreciative of the benefits of digital education, recognizing that abundant resources and diverse teaching methods could help students to grasp core knowledge more intuitively, thereby enhancing teaching effectiveness and efficiency. Yet this recognition was shadowed by their criticisms of resource quality—“the videos we shot are so poor in quality that students do not want to watch them”(T5). Another participant noted that “current online resources remain teacher-centred and fail to reflect learner-centredness” (T9). For these program directors, therefore, the experience of ‘teaching enhancement’ was permeated with an internal contradiction: they largely embraced the possibilities of technology, yet harboured unspoken doubts about its actual educational efficacy.

“Digital transformation enables students to grasp core knowledge more profoundly through online resources and 3D visualization techniques and aids teachers in enhancing teaching effectiveness and efficiency” (T1).

“Digital resources can reform our teaching mode and enrich teaching methods. But I don’t think the quality is really good... the videos we shot are so poor in quality that students do not want to watch them” (T5).

“Students can learn through online learning platforms or some APP programs according to their own learning progress and needs...which may improve learning efficiency and quality to a certain extent” (T9).

#### 3.1.2. Freedom and loss of control.

Digital education has transcended the temporal and spatial constraints of traditional teaching, enabling students to learn anytime and anywhere, unbound by fixed class schedules or physical classrooms. Yet this expanded reach came at an unforeseen cost: as instruction spilled into students’ private spaces, program directors simultaneously lost their sense of pedagogical control. One participant recalled a particularly vivid pandemic-era teaching experience: “Some students lay in bed, sleeping, while the lecture played on their phones” (T13). The technology that was supposed to enhance teaching instead left them trapped in anxious uncertainty. This entanglement of freedom and loss of control constituted the core tension in their digital education experience.

“Digital transformation enables students to access a wider range of learning resources and follow a more diverse array of learning paths, breaking the constraints of time and space” (T6).

“Digitalization enables open online teaching, providing students with opportunities for online learning and breaking through geographical and temporal limitations...But for children with poor self-control, we may feel a sense of powerlessness during the supervision process” (T12).

#### 3.1.3.  Student engagement.

Program directors generally believed that digital education, through videos, animations and interactive software, could significantly enhance students’ interest and participation and promote active learning. Yet some remained deeply skeptical about the authenticity of this “engagement”, noting that they had “no good way to detect whether students were really using it” (T8).Other participants revealed this uncertainty from the perspective of academic misconduct: students engaged in “copy-paste assignments” and “cheating on exams” (T13), using technology to bypass learning while platform monitoring functions could not fully prevent such behaviors. Program directors thus experienced a paradox: they observed increased classroom participation and had access to extensive learning data, yet whether students truly internalized knowledge remained opaque. Thus, fostering autonomous and deep learning emerged as a central tension in the digital transformation of nursing education.

“Digital teaching provides a richer and more convenient learning experience, enhancing students’ interest in learning and their level of participation” (T12).

“Digital technology can increase some of our students’ enthusiasm and participation in learning...But when using virtual simulations, students weren’t actually using their brains; they were just following the program” (T8).

“If I post teaching tasks on the platform, there may be copying and pasting, as well as exam cheating, when they are completed”(T13).

### 3.2.  Perceived status and impact of digital infrastructure

#### 3.2.1.  Resource development.

Most participants mentioned the construction of digital resources, and many efforts have been made to develop online courses, virtual simulation laboratories, and digital textbooks.However, interview data revealed a critical tension between the scale of construction and effectiveness of integration. While many “quality courses” and platforms exist, participants concurrently pointed to issues with siloed development and low utilization; one participant even admitted that they did not like to go back and watch the courses they had recorded (T14), suggesting that the mere existence of resources does not guarantee their educational impact or efficient use.

“In recent years, I have felt that digital development... is a bit fragmented, with each person doing their own resources... which has resulted in a significant waste of human, material, and financial resources.”(T6).

“The majority of our school’s courses are being developed into online open quality-courses, with teaching materials incorporating digital resources such as MOOCs, e-books, and videos” (T13).

“The school establishes an Information Center...and offers two national- and provincial-level online quality courses...We also recorded some online classes ourselves, but after finishing them, we found that we didn’t really like them and went back to watch them”(T14).

#### 3.2.2.  Infrastructure status.

The school has invested significantly in the construction of both hardware and software facilities, including establishing smart classrooms, equipping them with virtual simulation devices, and upgrading the network infrastructure.Yet, descriptions often focused on specifications and cost, with less detail on routine pedagogical integration. Furthermore, access to advanced equipment such as high-fidelity simulators was described as limited and often reserved for top students or special training—a reality that suggests such facilities were perhaps never intended for routine teaching. The pursuit of cutting‑edge technology has thus run counter to the principle of universal access, highlighting a gap between procurement and equitable, practical application for all learners.

“The school is constructing a digital immersive experience pilot base, covering an area of 1000 square meters, with a cost of 9.7 million, including VR and AR equipment” (T3).

“Our virtual simulation classroom, we have regular classes of seventy or eighty students—and that room, it simply cannot hold that many people” (T11).

“We have high-fidelity simulators,but because its model is very expensive, so student hands-on opportunities are not particularly maybe only the best-performing student gets to practice on it...but most students cannot practice it” (T8).

#### 3.2.3.  Integrate digital resources into lectures.

The incorporation of digital resources into theoretical instruction has become increasingly prevalent, predominantly adhering to a structured hybrid model that encompasses pre-class, in-class, and post-class activities. Various platforms are employed for the distribution of materials, administration of quizzes, facilitation of discussions, and collection of assignments. This signifies a methodical digitization of conventional teaching workflows, with the objective of extending learning opportunities and providing data for formative assessment.However, some participants also expressed that some teachers would consider returning to traditional classrooms and attending classes without mobile phones to improve teaching quality. This reflects the tension between teachers’ cognition and their mastery of digital technology.

“Corresponding digital resources are available before, during, and after class, utilizing the portable classroom and the intelligent vocational education platform” (T1).

“Prior to the class, a chapter preview is released that includes ideological and political elements of the course. After class, questions and assignments are posted, online tests are conducted, and feedback is provided” (T13).

“Perhaps some teachers, considering the issue of teaching quality... will still return to traditional classroom use. We call it ‘Phone parking lot’, and they specifically put away students’ phones so that they can go to class without phones”(T13).

#### 3.2.4.  Application in practical teaching.

In practical training, digital tools—particularly virtual simulation—are utilized to establish safe and repeatable environments for the development of clinical skills. This approach is widely regarded as essential for bridging theoretical knowledge with practical competence, especially in high-stakes or complex procedures. However, its efficacy remains a subject of debate among program directors, who raise concerns about the fidelity and pedagogical value of virtual simulations compared to physical models or real-world practice, thereby perpetuating an ongoing discourse regarding the role of simulation in skill acquisition.

“The smart laboratory is utilized for experimental teaching and remote hospital interactions, whereas the virtual simulation laboratory is employed for the recording of advanced nursing courses” (T3).

“In our practical courses, many frontline teachers believe that using models yields better results than digital simulation,the experience can resemble a game-like activity.I feel it’s not as effective as using a model.” (T11)

“The virtual simulation training room is utilized for training in venipuncture, during which students first practice in a virtual environment before proceeding to the practical training room for operation” (T14).

### 3.3. Systemic tensions and barriers

#### 3.3.1.  Policy practice gap.

The gap between high-level policy support and tangible teaching outcomes is a fundamental systemic tension. Although leaders recognize the need for significant financial investment and institutional commitment to develop digital courses and infrastructure, these inputs often do not translate into high-quality, readily usable teaching resources. In practice, digital resources are often described as “abundant but of low quality,” with inconsistent standards that fail to engage students or meet sophisticated teaching needs. Moreover, many of these resources remain underused, leading to a perception of “resource wastage.”Program directors thus found themselves caught between the imperative to secure resources from above and the subsequent reality of teacher resistance, technical obstacles, and student disengagement on the ground—a weary struggle of navigating between policy demands and ground-level resistance.

“I go to the leaders every day, sitting there or waiting by the door... I use key performance indicators and our contribution to the high-quality assessment to persuade them” (T2).

“Although the school possesses a wealth of digital resources, some teachers and students fail to fully utilize them, resulting in a waste of resources” (T2).

“Another thing is the school‘s supporting policies... for example, I spent so much energy and physical strength to build the course... so I don’t get any reward either” (T6).

“The quality of digital resources is problematic... Some resources, although sourced from various sources, exhibit inconsistent quality” (T14).

#### 3.3.2.  Readiness divide.

A significant barrier to implementation is the pronounced readiness divide, which encompasses varying levels of digital competence among educators and differing degrees of acceptance and autonomy among students. On the part of educators, this divide reflects not only skill gaps but also a deeper tension between external mandates and internal motivation or identity. This observation highlights a critical distinction: the central issue is not whether teachers and students “know how to use” digital tools, but rather whether they are “willing to use” them. At the same time, students often demonstrate low levels of initiative and engagement with digital resources, while challenges associated with self-regulation further hinder effective participation. As a result, program directors must navigate a delicate balance between administrative authority and professional empathy—caught between the imperative to mandate change and the need to acknowledge the genuine struggles of those they lead.

“For older teachers, using new platforms feels very resistant... whereas younger teachers learn quickly...I think teachers of different ages...we need to keep learning...” (T2).

“Teacher-related factors are a significant influencing factor… The most important one is the teacher’s own perception... He or she doesn’t realize we have entered a digital era” (T5).

“Students’ insufficient self-control leads to a tendency to be distracted when using digital platforms, making it difficult for them to focus on their studies” (T6).

“When it comes to teaching ability competitions... some teachers may think that you use so much, such as live streaming and having classmates post bullet comments on site... They think it’s fancy and worthless. Young teachers will definitely think that this form is good and can be applied in trial classes” (T11).

#### 3.3.3.  Technical operational barriers.

The usability and reliability of the digital ecosystem present substantial operational barriers that directly impede teaching. The compatibility and stability of technology platforms are critical, yet program directors frequently encounter issues such as unstable networks, user-unfriendly software, and fragmented systems that force data to be manually reentered across platforms. These technical shortcomings force both teachers and students to expend considerable effort on troubleshooting and adaptation rather than focusing on pedagogical content, thereby directly contradicting the promise of efficiency and creating tangible resistance rooted in daily operational friction.

“Network factors are significant hardware issues... At our old campus, because the network base station distribution was sparse, the signal could be poor, especially on the first floor, which affected our in-class testing” (T1).

“The platforms are not interconnected... For example, the exam system scores need to be manually re-entered into the academic system... They are not synced” (T6).

“Some software is indeed not very user-friendly, and sometimes the internal network is not very convenient... Sometimes it is difficult for us to log in... I hope it becomes more user-friendly and convenient to use, the more stupid the better” (T7).

“Some software platforms are not user friendly... It’s not as simple as scanning a WeChat code to view content; there are many cumbersome steps, which makes them impractical” (T11).

#### 3.3.4.  Workload pressures.

Digital transformation introduces unintended workload pressures, adding layers of complexity and burden for both students and educators. A key concern is the perceived imbalance between an increased time and effort investment and unclear returns in teaching effectiveness. Program directors report that developing digital resources significantly increases their pre- and post-class workloads; the use of digital platforms before and after class adds an invisible yet substantial layer of work. Concurrently, program directors note and must manage the cognitive overload students experience from multiple, uncoordinated digital demands across different courses, resulting in student fatigue and disengagement.

“Students are overwhelmed... Every course uses online platforms, so students feel burdened with too many tasks... We need to coordinate and find a balance” (T1).

“Teachers are too busy... There’s also teacher burnout... and a lack of interest” (T6).

“Things that initially increase the workload, if the online courses are well developed, should ultimately reduce it, but that hasn’t been achieved yet” (T8).

#### 3.3.5.  Professional role dissonance.

Beyond tangible resource and workload issues, digital transformation triggered a crisis of professional identity among program directors. They found themselves balancing their core roles as clinical mentors with a new set of technically driven demands. This dissonance stemmed from a dual pressure: deep skepticism about the pedagogical value of certain digital tools that seemed to undermine the hands-on, relational core of nursing and the unwanted expansion of their role into areas such as content creation and technical management, for which many felt unqualified. Caught between perceived threats to their teaching values and the imposition of alien responsibilities, they experienced an erosion of professional agency and motivation, creating a significant internal barrier to embracing digital innovation.

“We are, after all, not professional technical staff... Our teachers... may need the help of technical personnel,... but many schools may not have such dedicated personnel to assist our full-time teachers” (T1).

“The procurement procedures, including drafting contracts... all had to be done by the specialized teachers themselves... When faced with these additional, unfamiliar tasks, they become apprehensive and simply preferred not to purchase new equipment” (T5).

“I am also a beginner this year... because I have been managing student skill competitions. But when it comes to digital education skills, it's a completely different field, and I need to learn more” (T2).

### 3.4.  Strategic approaches to digital transformation

#### 3.4.1.  Infrastructure investment.

The call for increased investment transcends mere budget appeals, reflecting a strategic recognition that infrastructure is the nonnegotiable substrate of digital integration. However, program directors frame this need not as a passive receipt of funds but as a targeted and sustainable endeavor. The emphasis lies on creating a resilient ecosystem—encompassing stable networks, user-friendly platforms, and dedicated support teams—that reliably underpins daily pedagogy rather than serving as a sporadic showcase.To secure the resources necessary for such ecological construction, program directors emphasized the importance of adopting an outcomes-oriented strategy, leveraging demonstrable results and performance indicators to advocate for sustained institutional investment.

“Our institute has established an information technology group, primarily tasked with supporting the development of information resources and constructing an online course framework” (T1).

“The school’s support for digital education is insufficient, owing to a lack of funding, necessitating an increase in financial support” (T10).

“Digital education indeed requires sufficient financial support, encompassing infrastructure development, the procurement of educational resources, and teacher training” (T14).

#### 3.4.2.  Educator development.

Proposals for teacher development transcend generic training mandates to address the core motivational and identity-based challenges revealed in the readiness divide. The suggested approach is twofold: systemic support through regular, accessible upskilling and the cultivation of internal communities of practice. Strategies such as leveraging “model teams” from successful projects or implementing mentorship systems highlight a move toward peer-driven, experiential learning that connects digital tools directly to pedagogical content and professional recognition, thereby aiming to convert apprehension into agency.

“Our information center regularly conducts introductions to and training sessions on new technologies and advancements for teachers in school, aiming to enhance their digital skills” (T2).

“Teachers’ personal factors are a significant influencing factor, necessitating extensive training and learning to enhance their digital literacy” (T5).

“The majority of teachers are currently in the exploratory phase, necessitating the establishment of a systematic training regime in the future to enhance their digital teaching capabilities” (T13).

#### 3.4.3.  Resource innovation.

The drive for resource innovation is critically framed against the prevailing reality of “fragmented construction” and waste. The advocated strategy is not simply to produce more resources but to incentivize quality and orchestrate sharing to break down silos. Doing so involves establishing mechanisms that reward the creation of contextually relevant, high-quality original materials while building platforms or agreements that facilitate their circulation. The goal is to transform the current paradigm from one of isolated, often redundant, production to a collaborative ecosystem, thereby enhancing collective efficacy and resource richness.

“The most critical aspect of digital empowerment is collaborative construction and shared utilization. The current issue is the prevalence of fragmented approaches, leading to a severe waste of resources” (T6).

“We need to enhance the quality of digital resources, encourage the development of original content, and facilitate resource sharing through platforms” (T14).

#### 3.4.4. Incentive mechanisms.

Discussions around incentive mechanisms underscore the need to formally recognize and value the additional labor inherent in digital transition. Program directors articulate the need for a multifaceted system that links performance in digital teaching to tangible outcomes in professional evaluation, promotion, and compensation. This need is complemented by calls for scientific assessment frameworks that move beyond superficial metrics to measure genuine teaching effectiveness and student learning. The overarching aim is to create a feedback loop in which effort in digital pedagogy is seen, measured, and rewarded, thus legitimizing it as core professional work.

“The school will provide corresponding policy support and incentives on the basis of the teachers’ digital teaching performance, thereby enhancing their enthusiasm” (T2).

“A scientific assessment mechanism should be established, encompassing student evaluations, teacher evaluations, and course evaluations, to continuously enhance the quality of digital teaching” (T4).

“Schools should have a relatively effective teaching performance system, which grants corresponding coefficient rewards to teachers who diligently implement digital teaching methods” (T13).

#### 3.4.5.  Industry–education integration.

Industry-education integration is conceived as a crucial channel for bridging the clinical–digital divide and enhancing the relevance of teaching resources. It is envisioned not merely as a form of equipment donation but also as a deep collaboration for codeveloping scenarios (e.g., mobile-based virtual systems) and content that reflects authentic clinical practice and emerging technologies (e.g., telemedicine integration). Moreover, partnerships with digital education platform providers are essential for refining the daily usability of these tools, alleviating technical friction, and thereby enhancing the overall experience for educators. This approach seeks to move digital resources from simulated generality to contextualized specificity, thereby increasing their utility for student learning and ensuring that educational transformation remains connected to the evolving realities of healthcare delivery.

“We are seeking collaboration with enterprises to develop a virtual teaching system that can be implemented on mobile phones, aiming to enhance students’ learning outcomes” (T1).

“Digital transformation can be integrated with telemedicine and teaching, enabling students to engage in video communications with frontline clinical experts” (T9).

## 4.  Discussion

This study aims to explore Chinese higher vocational nursing program directors’ perceptions and experiences regarding the digital transformation of education. We introduce the technology acceptance model (TAM) as the core theoretical framework. The TAM posits that perceived usefulness and perceived ease of use are critical factors influencing individuals’ attitudes and behavioral intentions toward technology adoption, which are moderated by external variables [18]. On the basis of this framework, we analyzed the interview data systematically, mapping program directors’ recognition of digital value, perceptions of implementation barriers, and recommendations to the dimensions of “perceived usefulness,” “perceived ease of use,” and “external variables” within the TAM. This approach not only provides theoretical support for advancing the digital transformation of higher vocational nursing education but also enables a comparative analysis with the global research on technology acceptance in nursing education.

### 4.1. Perceived usefulness

Nursing program directors generally acknowledge the value of digital education, demonstrating strong perceived usefulness. They believe that digital technologies can enrich teaching methods, expand learning time and space, and improve management efficiency, which aligns with global research findings [19,20]. The interview results indicate that the “usefulness” of digital technologies remains superficial and is limited to convenience and resource abundance, with insufficient deep integration into teaching objectives and learning processes. Some technologies were even perceived as less useful than anticipated [21]. Only when students genuinely perceive digital technologies as enhancing their clinical competencies will acceptance levels and educational effectiveness improve significantly [22]. Therefore, program directors should move beyond superficial “technological usefulness” by designing challenging and gamified clinical scenario simulations in subsequent classes [23,24], accompanied by classroom incentive mechanisms to increase students’ perceived usefulness [25]. Additionally, by leveraging big data and AI-driven personalized recommendations, educators can deliver tailored learning resources and tasks aligned with students’ interests and progress. This not only achieves seamless integration with teaching objectives and learning processes but also optimizes resource allocation, effectively preventing the waste of digital resources.[26]. Such an integration enables both educators and students to experience the practical value of digital teaching intuitively, realizing profound technological usefulness [27].

### 4.2. Perceived ease of use

Despite substantial global investments in digital education [28], the participants’ perceived ease of use remains suboptimal. First, the nursing program directors highlighted issues such as inconsistent resource quality, platform incompatibility, and lagging technical maintenance, forcing educators and students to expend energy managing technological uncertainties rather than focusing on pedagogy, a foundational barrier to perceived ease of use [29]. Second, varying levels of digital literacy among teachers constitute a critical factor affecting perceived ease of use [30]. Although high digital literacy enhances teaching effectiveness [31], some teachers exhibit low levels of learning motivation,compounded by insufficient institutional technical training and support, a pattern that is consistent with the findings of other studies [32]. Perceived ease of use and perceived usefulness often influence each other [33]; when platforms are difficult to operate, program directors tend to question their practical value [34]. Thus, in the AI era, teaching platforms should integrate large AI models to optimize interface layouts and software–hardware ecosystems [35,36], particularly VR systems, to ensure logical functionality [37,38]. Institutions should standardize teaching platforms and provide systematic training to alleviate technical anxiety [30,39]. Continuous feedback channels, such as community platforms [39], should be established during implementation to address practical challenges and form a virtuous cycle of training–application–feedback, ultimately enhancing platform usability.

### 4.3. External variables

External variables represent critical contextual factors influencing individual perceptions and behaviors [40]. National policy serves as a macrolevel external variable [41]. Although national-level initiatives strongly advocate for AI development, school evaluation and incentive mechanisms remain underdeveloped, a finding corroborated by similar studies. When additional efforts in digital teaching fail to receive formal recognition in professional title evaluation or performance assessments, program directors tend to strive to meet superficial indicators [42]. Therefore, establishing promotion and reward systems linked directly to digital teaching innovation outcomes is crucial for transforming policy directives into intrinsic motivation [43]. With respect to resource ecosystems, despite increased regional investments, fragmented platforms and dispersed resources force program directors to spend excessive amounts of time screening and adapting materials, increasing usage costs [44,45]. Although large language models such as ChatGPT and DeepSeek partially facilitate instructional design [46,47], their outputs require further verification because of limited access to professional educational resources [48,49]. Future government-led consolidation of authoritative resources into integrated platforms connected with AI models could liberate educators from technical burdens by aligning with user habits in lesson preparation, assignment distribution, and resource acquisition [50,51]. At the individual level, professional role dissonance emerged as a key internal factor mediating program directors’ acceptance of digital tools [52].Finally, student acceptance and engagement are equally vital. Vocational students often exhibit low enthusiasm for online learning tasks, with heavy course loads potentially causing resistance, reduced participation, and diminished self-efficacy [53,54]. Program directors should develop context-specific engagement strategies, ensuring that digital reforms prioritize the reduction of student burdens. The quality of digital interactions should surpass that of quantitative metrics [55].

Under the TAM framework, perceived usefulness, perceived ease of use, and external variables collectively influence nursing program directors’ acceptance and behavior toward digital technologies. These factors not only shape grassroots program directors’ and students’ attitudes toward digital transformation but also determine the bottom-up progression of the digitization of nursing education. Although this study is contextualized within China, its TAM-based recommendations and strategic framework offer actionable insights for digital transformation in similar vocational education environments globally.

## 5.  Conclusion

The study findings suggest that most higher vocational nursing program directors are supportive of digital education, as they recognize its perceived usefulness in enhancing teaching effectiveness and overcoming spatiotemporal barriers. However, as analyzed through the technology acceptance model (TAM), several key challenges remain, rooted in perceived ease of use and external variables. These challenges include ecosystem barriers such as an insufficient quality and integration of digital resources, inadequate technical support, and platform fragmentation, which collectively undermine ease of use, and systemic barriers such as the low utilization of resources, significant variations in student participation, and the lack of institutional incentives, which act as critical external variables constraining adoption.

To address these TAM-identified challenges effectively, a shift is needed from isolated interventions to a comprehensive, systemic strategy. Institutions must not only augment investment and upgrade infrastructure but also, crucially, consolidate and streamline their digital ecosystems while offering integrated support to substantially improve perceived ease of use. Moreover, robust incentive structures must be implemented, for example, by tying digital teaching performance to career evaluations and advancement opportunities, thereby converting external variables into powerful motivators. Concurrently, fostering collaborative partnerships between educational institutions and industry is key to ensuring the clinical relevance of digital resources, thus amplifying their perceived usefulness. The ultimate success of this endeavor depends on a concerted, multistakeholder effort that strategically targets all facets of technology acceptance—usability, motivation, and practical value—to elevate teaching standards and nurture the next generation of nursing professionals synergistically.

## 6.  Study limitations

This study has several limitations. First, the participant sample included nursing program directors who, while engaged in frontline teaching activities, hold administrative roles. Thus, the findings may not entirely capture the unique experiences of full-time, nonadministrative frontline educators and students. Future research should intentionally include these specific groups to achieve a more balanced and comprehensive understanding. Second, although qualitative interpretation can entail researcher bias, several strategies were implemented to enhance the trustworthiness of the data and analysis. These strategies included investigator triangulation through independent coding by two researchers, regular consensus discussions within the research team to refine themes, and the maintenance of an audit trail of analytical decisions. Despite these limitations, the study offers valuable insights for promoting digital transformation in vocational nursing education.

## Supporting information

S1 File Summary table of themes with illustrative quotes (Bilingual). (DOCX)
