# Supplementary material for: Perceptions of Chinese higher-vocational nursing program directors on digital transformation: A qualitative study
Source: PLoS One. 2026 Apr 8;21(4):e0346261. doi: 10.1371/journal.pone.0346261 (PMC13061177; doi:10.1371/journal.pone.0346261)
Supplement: S1 File — (DOCX) [file pone.0346261.s001.docx]

**3.1. Recognition of Digital Education by Program Directors**

**3.1.1. Teaching Enhancement**

| **Participant** | **Original Chinese Quote** | **English Translation** |
| --- | --- | --- |
| T1 | “数字化转型能够帮助我们...推动到学生可以通过网络资源，还有更立体的一些3D立体的一些手段，帮助他们能够更深刻的去掌握一些知识点。也帮助我们教师在教学上提高我们的教学效果，还有教学的效率。” | "Digital transformation can help us... by enabling students to utilize online resources and more three-dimensional methods to grasp knowledge points more profoundly. It also helps us teachers improve our teaching effectiveness and efficiency." |
| T2 | “数字化转型...那肯定必须要紧跟这种发展...教育的变革也会引起...高职护理教育的话，也必须跟着相应的做一些相应的变革...” | "Digital transformation... must definitely keep pace with this development... Changes in education will also drive... higher vocational nursing education must also undergo corresponding reforms..." |
| T3 | “数字化转型赋能高职护理教育教学发展，是下一步高职护理教育发展的必然方向之一，也是提高现有教育教学质量水平的必要...是将现在课堂由扁平化向立体化发展的方式...” | "Empowering higher vocational nursing education through digital transformation is an inevitable trend for its future development and is necessary for improving the quality of current education and teaching... It is a way to transform the current classroom from flat to three-dimensional..." |
| T4 | “数字化转型赋能...其实很多方面的话都可以应用到...从我们教师的培养，学生的培养，然后甚至一些社会服务各方面...几乎都能够去用数字化的这种去完成一些内容...” | "Empowerment through digital transformation... can actually be applied in many aspects... from the training of our teachers and students, to even various social services... almost all can be accomplished using digital means..." |
| T5 | “数字资源能够革新我们的教学模式并丰富教学方法。但我认为其质量并不理想……我们拍摄的视频质量极差，以致学生不愿观看。” | “Digital resources can reform our teaching mode and enrich teaching methods. But I don’t think the quality is really good... the videos we shot are so poor in quality that students do not want to watch them” |
| T9 | “学生可根据自身学习进度和需求，通过在线学习平台或部分APP程序进行学习……这在一定程度上可能提升学习效率与质量。” | “Students can learn through online learning platforms or some APP programs according to their own learning progress and needs...which may improve learning efficiency and quality to a certain extent” |
| T10 | “数字化的转型确实对我们高职护理教育发展起到了非常正向的促进的作用...我们在利用这种信息化的一个资源，学生可以学到顶尖的这种学校的这样的一个授课的内容。老师也可以同样学习到其他的一些很前沿的一些知识和内容，利于自己知识的一个更新。” | "The digital transformation has indeed played a very positive role in promoting the development of our higher vocational nursing education... By utilizing these informational resources, students can access teaching content from top-tier schools. Teachers can also learn cutting-edge knowledge and content from others, which helps update their own knowledge." |
| T12 | “数字化转型对高职护理教育具有很重要的赋能作用。因为它是我们传统教学的一个补充，并且它有非常多的优点...它提升了学生的学习的体验...更丰富的、更便捷的、多样化的学习体验...更直观的学习和理解知识，增强学习兴趣和参与的程度。同时我们老师也可以通过这个数据分析来评估和改进教学的策略。在很大程度上它提高了教学效果。” | "Digital transformation plays a very important empowering role in higher vocational nursing education. Because it is a supplement to our traditional teaching and has many advantages... It enhances students' learning experience... providing a richer, more convenient, and diversified learning experience... enabling more intuitive learning and understanding of knowledge, increasing interest and participation. At the same time, we teachers can also use this data analysis to evaluate and improve teaching strategies. To a large extent, it improves teaching effectiveness." |
| T13 | “互联网...对我们教育教学的发展是起到一个很大的推进作用的...在很大一程度上把老师们这种他的时间，还有就是他的那种能力给凸显出来，把时间也解放了出来...对于学生来说，他们可能通过这样一个方式去学习，应该会有更多的自主性，甚至是自由性。” | "The Internet... has played a significant role in promoting the development of our education and teaching... It greatly highlights teachers' capabilities and liberates their time... For students, learning through this method might give them more autonomy and even freedom." |
| T14 | “目前的这个数字化的教学应该是成为教育的一个主要趋势。数字化转型它可以赋能各层次的教育...可以给学生提供比较优质的教育资源，而且更加能够贴近实际的实践教学体验。” | "Current digital teaching should become a major trend in education. Digital transformation can empower all levels of education... can provide students with relatively high-quality educational resources and offer practical teaching experiences that are closer to reality." |

**3.1.2. Freedom and Loss of Control**

| **Participant** | **Original Chinese Quote** | **English Translation** |
| --- | --- | --- |
| T3 | “是将基于课堂的教学延伸向不同地域、不同空间、不同时间人群的一种方式。并且数字化手段...可以完成现在课堂教学当中无法完成的一些情况。比如说利用课堂手段与医院进行临床远程教学互动。再比如说。在不同的院校之间开展横向的教育教学联合...” | "It is a way to extend classroom-based teaching to people in different regions, spaces, and times. Furthermore, digital means... can accomplish things that are currently impossible in classroom teaching. For example, using classroom means to conduct remote clinical teaching interactions with hospitals. Another example is carrying out horizontal educational and teaching collaborations between different institutions..." |
| T4 | “在疫情期间也有线上线下教学...学生呢在线上学习的时候，他的这个主动性、自觉性是不足的...” | "During the pandemic, there was both online and offline teaching... when students studied online, their initiative and self-discipline were insufficient..." |
| T6 | “数字化转型让学生能够接触到更丰富多样的学习资源，走更多元化的学习道路，不再受时间和空间的限制啦...” | “Digital transformation enables students to access a wider range of learning resources and follow a more diverse array of learning paths, breaking the constraints of time and space” |
| T12 | “数字化技术推动了开放在线教学的发展，为学生提供了在线学习的机会，突破了地理和时间的限制……但对于自我控制能力较差的儿童，在监督过程中我们可能会感到无力感。” | “Digitalization enables open online teaching, providing students with opportunities for online learning and breaking through geographical and temporal limitations...But for children with poor self-control, we may feel a sense of powerlessness during the supervision process” |
| T13 | “有些学生躺在床上睡觉，而讲座在他们的手机上播放。” | “Some students lay in bed, sleeping, while the lecture played on their phones” |

**3.1.3. Student Engagement**

| **Participant** | **Original Chinese Quote** | **English Translation** |
| --- | --- | --- |
| T1 | “（学生）可以增加在利用手机或者是这种叫做颗粒化的时间，能够提升他们自主学习的一个积极性了。” | "(Students) can increase their utilization of fragmented time, such as with their phones, which can enhance their initiative for self-directed learning." |
| T5 | “这些（数字化手段）在上课过程用的时候，还都可以非常很好的调动学生的积极性，让上课的氛围变得特别的好。” | "When these (digital methods) are used during class, they can very effectively stimulate students' enthusiasm and make the classroom atmosphere particularly good." |
| T8 | “数字技术能够提升部分学生的学习热情与参与度……但当使用虚拟模拟时，学生并未真正动用大脑，而只是机械地遵循程序指令。” | “Digital technology can increase some of our students’ enthusiasm and participation in learning...But when using virtual simulations, students weren’t actually using their brains; they were just following the program” |
| T12 | “（数字化）增强学习兴趣和参与的程度...我们通过在网上的互动，比如说在线的讨论头脑风暴，抢答...还有像学习心得分享，自己的操作视频的上传。通过这些互动交流和合作，可以增强学生的学习效果。” | "(Digital means) enhance learning interest and the degree of participation... Through online interactions, such as online discussions, brainstorming, quick-response Q&A... and sharing learning insights, uploading their own operation videos. These interactive exchanges and collaborations can enhance students' learning outcomes." |
| T13 | “若我在平台上发布教学任务，待其完成后，可能会出现抄袭粘贴及考试作弊行为。” | "If I post teaching tasks on the platform, there may be copying and pasting, as well as exam cheating, when they are completed" |
| T14 | “目前我们在实施一些数字化或信息化教学的时候，学生还是参与度比较高的。” | "Currently, when we implement some digital or informational teaching, student participation is relatively high." |

**3.2. Perceived Status and Impact of Digital Infrastructure**

**3.2.1. Resource Development**

| **Participant** | **Original Chinese Quote** | **English Translation** |
| --- | --- | --- |
| T1 | “我们的数字化资源，还有数字化软硬件的建设，都是依靠在精品课程的一个建设平台上面做...我们依靠的主要的数字化资源的体现平台是我们自己学校有一个独立的一个平台，它叫做随身课堂。然后还有一个平台，就是我们会用智慧职教平台来建设我们的精品课程...我们有一本国家叫做规划教材，也是是属于数字化教材。也有这个数字化教材在建设...” | "The construction of our digital resources, as well as digital software and hardware, relies on a platform for developing high-quality courses... The main platforms where our digital resources are embodied are our school's own independent platform called 'Portable Classroom', and another platform, ZhiJiao (Wisdom Vocational Education), which we use to build our high-quality courses... We have one national 'planning textbook', which is also a digital textbook. We also have other digital textbooks under construction..." |
| T2 | “数字化资源的话，一方面...可能最多的还是一些针对相当于比如说像课程平台...这几年...尤其是疫情催生了很多...在线课程的建设...我们基本上用学习通，然后还有大学生慕课...”“我们有一些，我们觉得这边不合适，不方便我们要怎么调整，他们（超星）也会给我们加模块...”“数字化教材...我像以前也参加了两本...十三五的一个国规教材的一个这样的一个数字化教材的这样的一个编写工作...包括活页式教材...” | "Regarding digital resources, on one hand... probably the most are things like course platforms... in recent years... especially the pandemic has spurred a lot of... online course construction... We basically use Learning Through (Chaoxing), and also University MOOC...""We have some instances where we feel something isn't suitable or convenient, and how we want to adjust it, they (Chaoxing) will also add modules for us...""Digital textbooks... I previously participated in two... the compilation of digital textbooks that were part of the '13th Five-Year Plan' national planning textbooks... including loose-leaf textbooks..." |
| T3 | “我们正在建马上完工的一个数字化的沉浸式体验试验品基地...造价970万...其中有这个数字化产品的造价六百多万，牵扯到VR设备、AR设备...有一套虚拟仿真的实训室...解剖学的数字化教学式，还有那个数字化录播室...” | "We are building a digital immersive experience pilot base that is nearing completion... costing 9.7 million yuan... of which over 6 million is for digital products, involving VR equipment, AR equipment... There is a virtual simulation training room... a digital anatomy teaching lab, and that digital recording and broadcasting room..." |
| T4 | “我们学校就是虚拟仿真，我们有一个医学虚拟仿真中心，初步已经建成了...可以说是全国第一家做的这么快这么早的...”“我们的课程建设有很多是在线上的，就是利用那个学习通职教云，甚至还有一些叫助微助教，雨课堂等等。” | "Our school has virtual simulation; we have a Medical Virtual Simulation Center that has been initially built... it can be said to be the first in the country to do it so quickly and so early...""Much of our course construction is online, using platforms like Learning Through (Chaoxing) ZhiJiao Cloud, and even some called WeChat Assistant, Rain Classroom, etc." |
| T6 | “近年来，我注意到数字化发展...呈现碎片化态势，各方各自为政...导致人力、物力和财力资源严重浪费。” | "In recent years, I have felt that digital development... is a bit fragmented, with each person doing their own resources... which has resulted in a significant waste of human, material, and financial resources." |
| T8 | “我们省级的只有一个，就是我们的外科护理学是省级的线上精品课程。然后其他的话，它都是校内就是校级的精品课程...我们现在是要求每一个核心课程全部都是校级的精品课程...”“我们有那个试题库习题库...” | "We only have one at the provincial level, which is our 'Surgical Nursing' provincial-level online quality course. For the others, they are all school-level quality courses within the university... We now require that every core course must be a school-level quality course...""We have that test question bank, exercise bank..." |
| T9 | “我们护理学院有一门儿国家精品在线课程，还有七门省级精品在线课程，十几门的校级精品在线课程...现在我们学校的教材编写中基本上都有二维码，有思政的内容，有课后习题，另外还有一些操作的视频供学生观看。” | "Our nursing school has one national-level quality online course, seven provincial-level quality online courses, and over ten school-level quality online courses... Now, in our school's textbook compilation, they basically all include QR codes, contain ideological and political education content, have post-class exercises, and also some operation videos for students to watch." |
| T10 | “老师们在上课的时候...学校里面也有数字平台，老师们会在平台上建课...老师们上课可以说几乎无一例外的都在使用平台。有的使用学习通，有的有语课堂，什么微助教，还有我们的智慧职教...”“课程的建设的质量说实在话还不是特别好。老师们可能顶多就是放一些课件上去...” | "When teachers are in class... the school also has a digital platform, teachers build their courses on the platform... It can be said that almost without exception, teachers are using the platform in class. Some use Learning Through (Chaoxing), some use Rain Classroom, something called WeChat Assistant, and our Wisdom Vocational Education (ZhiJiao)...""To be honest, the quality of course construction is not particularly high. Teachers might at most upload some courseware..." |
| T11 | “我们学校现在有那个省级精品课程，正在申请国家级精品课程...我们的教材是校本教材...而且是校企联合编的...要求这个使用率和点击率...教材的章节，还有他的思维导图，还有这一章的重点难点提示...其实他是在我们的建设课程里面...有知识拓展一般都是以微课的形式出现的。然后接下来就是他的复习题，本章复习题，本章重难点提示，然后还有就是思维导图都在。” | "Our school now has that provincial-level quality course and is applying for a national-level quality course... Our textbooks are school-based textbooks... and are co-compiled with enterprises... requiring usage rate and click-through rate... The textbook chapters, along with their mind maps, and the key and difficult point prompts for the chapter... are actually within our constructed course... Knowledge expansion generally appears in the form of micro-lectures. Then comes its review questions, chapter review questions, chapter key and difficult point prompts, and then the mind maps are all there." |
| T12 | “现在我们建设的校级的数字资源库，有一个校级的精品在线开放课程，一共是二十九门。而省级的精品在线开放课程有五门，并且建设了一个虚拟仿真实训基地...” | "Now we have built a school-level digital resource library, with school-level quality online open courses, totaling twenty-nine. There are five provincial-level quality online open courses, and a virtual simulation training base has been built..." |
| T13 | “大部分的课程其实都在想办法以这种在线开放的精品课程为目标去做这样一个课程建设...老师们所参编的一些教材，都肯定会包含有一些数字化的资源...比如说数字化资源里面有慕课，有电子书，有一些视频...”“我们在学生大三的时候...用超星平台给他们进行一个期中考试或者是期末考试...一个课程所建设的题库可能就会达到5000道左右...” | "Most courses are actually trying to aim for this type of online open quality course as a goal for their course construction... Textbooks that teachers participate in compiling will definitely include some digital resources... For example, among the digital resources, there are MOOCs, e-books, some videos...""When students are in their third year... we use the Chaoxing platform to conduct midterm or final exams for them... the question bank built for one course might reach about 5,000 questions..." |
| T14 | “我们学校设置了这个信息中心...负责学校的智慧校园管理平台技术保障，负责学校综合教学平台的技术保障...负责课程在线开放课程的组织建设工作。负责学校的教学库的组织资源的建设工作...”“我们临床护理学院...建有一个虚拟仿真实训室，虚拟仿真静脉穿刺实训室...还有一个就是在基础医学院...有一个生命科学馆，还有一个是数字化的人体解剖模型...护理系的这个实训中心，然后设置了一个模拟医院...”“教学资源库的建设，我们目前是由两门省级的教学资源，两门省级的这个在线精品课...一门国家级的在线精品课...这个在线精品课今年又申报了四项...我们自己也录过一些网课，但看完后发现其实不太喜欢，就又回来看了一遍。” | "Our school has established an Information Center... responsible for the technical support of the school's smart campus management platform, responsible for the technical support of the school's comprehensive teaching platform... responsible for the organization and construction of online open courses. Responsible for the organization and resource construction of the school's teaching repository...""Our Clinical Nursing College... has built a virtual simulation training room, a virtual simulation venipuncture training room... Also, in the Basic Medical School... there is a Life Science Museum, and a digital human anatomy model... The training center of the Nursing Department has set up a simulated hospital...""For the construction of the teaching resource library, we currently have two provincial-level teaching resources, two provincial-level online quality courses... one national-level online quality course... We have applied for four more online quality courses this year......We also recorded some online classes ourselves, but after finishing them, we found that we didn’t really like them and went back to watch them" |

**3.2.2. Infrastructure Status**

| **Participant** | **Original Chinese Quote** | **English Translation** |
| --- | --- | --- |
| T1 | “我们学校现在是国家的虚拟仿真系统的一个中心，有一个国家级的一个中心，我们也有一个独立的管理数字化教学的一个部门，叫做信息中心。” | "Our school is now a national center for the virtual simulation system, having a national-level center. We also have an independent department managing digital teaching, called the Information Center." |
| T2 | “硬件方面的话，基本上现在像一些虚拟仿真...高仿真的...高端的模拟人，我们大概有三台...另外还有一个像...sam baby...same mom...还有一些智能性的一些交互式的一些老年的一些...虚拟穿刺，什么临床思维系统...”“我们准备搞智慧病房...申请500万准备搞智慧病房...” | "In terms of hardware, basically now things like virtual simulation... high-fidelity... high-end patient simulators, we have about three... Also, there are things like... SimBaby... SimMom... and some intelligent interactive ones for geriatrics... virtual puncture, clinical thinking systems...""We are preparing to build a smart ward... applied for 5 million to build a smart ward..." |
| T3 | “我们正在建马上完工的一个数字化的沉浸式体验试验品基地。整个实训基地占地1000平米，造价970万。其中有这个数字化产品的造价六百多万，牵扯到VR设备、AR设备以及大量的沉浸式体验的数字化互动设备...虚拟仿真的实训室...解剖学的仿真实验室...数字化录播室...智慧教室已经建设完成...正在启用智慧化实验室。智慧化实验室包括门禁系统、门牌系统、自动二次排课系统以及实验室资产管理系统和预约实验系统。还有一个就是项目类考试系统...” | "We are building a digital immersive experience pilot base that is nearing completion. The entire training base covers an area of 1000 square meters, costing 9.7 million yuan. Over 6 million of that is for digital products, involving VR equipment, AR equipment, and a large amount of digital interactive equipment for immersive experiences... virtual simulation training room... anatomy simulation lab... digital recording and broadcasting room... smart classrooms have been completed... smart laboratories are being commissioned. The smart laboratories include access control systems, doorplate systems, automatic secondary course scheduling systems, laboratory asset management systems, and experiment booking systems. There is also a project-based examination system..." |
| T4 | “我们学校就是虚拟仿真...建了第一期。第二期...比它更先进...还有一些基础实训室，也有一些模型人的那种智能化，甚至一个虚拟的静脉输液装置，还有一些老年人的一些智能化的东西...” | "Our school has virtual simulation... built the first phase. The second phase... is more advanced than it... There are also some basic training rooms, also some intelligent manikins, even a virtual intravenous infusion device, and some intelligent equipment for the elderly..." |
| T5 | “数字化软硬件建设方面，我觉得我们学校应该是还可以，相关的一些硬件、软件购买的都还行，不是特别特别好，但是也不是特别差，属于中等水平。比如说像分娩妊娠分娩的虚实结合，然后还有比如说在心肺听诊里面的，我们有购买了...心肺听诊系统。” | "Regarding the construction of digital software and hardware, I think our school should be okay. The relevant hardware and software purchased are fairly good, not particularly excellent, but not particularly bad either, belonging to a medium level. For example, the virtual-real combination for childbirth and pregnancy, and then, for instance, in cardiopulmonary auscultation, we have purchased... a cardiopulmonary auscultation system." |
| T7 | “我们学校也投入了不少的钱去建设软件和硬件。硬件的话目前就是多媒体教室，还有这些多媒体的机器，一般都上课都能正常的使用。但是维护就可能有时候跟不上...” | "Our school has also invested a lot of money in building software and hardware. In terms of hardware, currently there are multimedia classrooms, and these multimedia machines, generally they can be used normally in class. But maintenance sometimes might not keep up..." |
| T8 | “我们拥有高保真模拟器，但由于其模型成本高昂，学生实际操作的机会极为有限，可能仅有表现最优异的学生得以进行实践……而大多数学生无法获得此类实践机会。” | "We have high-fidelity simulators,but because its model is very expensive, so student hands-on opportunities are not particularly maybe only the best-performing student gets to practice on it...but most students cannot practice it." |
| T9 | “我们学校也在努力的进行数字化转型，但是目前硬件和软件都有待提高...我们可能推行数字化教育方面，主要是在教学课堂中使用了一些智能白板、投影仪...另外有线上的虚拟实验的软件和设备...我们还建了在线学习平台...在临床实训方面，我们目前虚拟实验室还在建设中。但是我们健康评估、急救等确实有虚拟的模拟人体模型...我们附属医院还有远程的这种模拟医疗系统...” | "Our school is also striving for digital transformation, but currently both hardware and software need improvement... In promoting digital education, we mainly use some smart whiteboards, projectors in the teaching classroom... Also, there are online virtual experiment software and equipment... We have also built an online learning platform... In terms of clinical training, our virtual laboratory is still under construction. But we do have virtual simulation manikins for health assessment, emergency care, etc.... Our affiliated hospital also has a remote simulation medical system..." |
| T10 | “我们的护理...专业开办的时间也不是很长...所以目前我们的数字化资源软硬件建设相对来讲是比较滞后的...虚拟仿真软件，我们都很少有有的非常少...” | "Our nursing... program hasn't been established for very long... so currently, our construction of digital resource software and hardware is relatively lagging... We have very few virtual simulation software, very few..." |
| T11 | “硬件方面现在我们学校硬件不太行...我们的虚拟仿真有有...虚拟仿真系统买的...厂家配了笔记本...平板...”“我们虚拟教室的常规班级通常有七八十名学生，但那个房间根本容不下这么多人。...” | "In terms of hardware, now our school's hardware is not great... We do have virtual simulation... The virtual simulation system we bought... the manufacturer provided laptops... tablets...""Our virtual simulation classroom, we have regular classes of seventy or eighty students—and that room, it simply cannot hold that many people..." |
| T12 | “建设了一个虚拟仿真实训基地，一个建成了这个智慧教室，一共是五间。” | "Built one virtual simulation training base, and built these smart classrooms, five rooms in total." |
| T14 | “我们学校设置了这个信息中心...独立的一栋楼...负责学校的网络的这种保障，还有负责学校的多媒体的维护...”“我们临床护理学院整个的基地的建设面积是26000平方米...设了全套监控设备的客观结构化考核...教学科研仪器价值是六千多万...”“虚拟仿真实训室...生命科学馆...数字化的人体解剖模型...模拟医院...”“智慧校园管理平台...” | "Our school has established this Information Center... a separate building... responsible for the school's network support, and also responsible for the maintenance of the school's multimedia...""Our Clinical Nursing College's entire base construction area is 26,000 square meters... equipped with objective structured clinical examination (OSCE) stations with full monitoring equipment... The value of teaching and research instruments is over 60 million yuan...""Virtual simulation training room... Life Science Museum... digital human anatomy model... simulated hospital...""Smart campus management platform..." |

**3.2.3. Integrate Digital Resources into Lectures**

| **Participant** | **Original Chinese Quote** | **English Translation** |
| --- | --- | --- |
| T1 | “在教学各个环节上面，我们包括课前、课中、课后都会有相对应的一些数字化资源...课前、课中和课后各项活动...根据智慧职教的这个平台来增加我们的数字化资源在里面。” | "Across all teaching stages, we include corresponding digital resources for pre-class, in-class, and post-class... various activities in pre-class, in-class, and post-class... We add our digital resources based on the ZhiJiao platform." |
| T2 | “课前布置作业...课前的作业我们从一开始的选择题到后面以后我们有一些比如填空、问答尤其是课后...然后因为算平时成绩...课前的作业...课前的任务清单...学生预习了以后...课堂上，我们主要是以翻转课堂...” | "Assign homework before class... the pre-class homework evolved from just multiple-choice questions at the beginning to later including things like fill-in-the-blanks, Q&A, especially after class... and because it counts towards usual grades... pre-class homework... pre-class task list... after students preview... in the classroom, we mainly use the flipped classroom model..." |
| T4 | “课前课中，我们这个点名，都会课后发布作业，都会用到咱们的平台...在上课的时候...给学生布置一些是拍摄视频...做成情景模拟的形式...还有的同学更高级点，他就自己制作了一些小动画...课前的点名，课中的这个小测课后的这个作业等等，这些都应用到了。” | "Pre-class, in-class, our roll call, post-class homework assignment, all use our platform... During class... assign students tasks like shooting videos... making them into scenario simulations... some more advanced students even create their own small animations... Pre-class roll call, in-class quizzes, post-class homework, etc., all these are applied." |
| T5 | “我自己会经常用雨课堂，用投屏系统，用一些小游戏。我觉得这些在上课过程用的时候，还都可以非常很好的调动学生的积极性...” | "I myself often use Rain Classroom, screen mirroring systems, and small games. I think when these are used during the class process, they can very effectively mobilize students' enthusiasm..." |
| T7 | “PPT的话在理论的这个教学上面，是发挥了比较大的作用的...课前课后课中的练习和讨论，什么头脑风暴，这些都在上面（智慧职教），作用还是比较多的。” | "PPT plays a relatively significant role in theoretical teaching... Exercises and discussions before, after, and during class, things like brainstorming, are all on there (ZhiJiao), and they are quite useful." |
| T8 | “课前发布习题，然后让他微课...我们会有一个思政导入...在课前的时候就会运用线上的一些资源，对它进行一个课程思政。课中的话...像学习通里面的它的点名、抢答，然后我们还有小组作业，小组汇报...课后的话也是一样的，就课后习题，然后课后上传他们的一些比较特别的东西...” | "Release exercises before class, then have them watch micro-lectures... We will have an ideological and political introduction... Before class, we use online resources to integrate curriculum ideology and politics. During class... things like roll call, quick-response Q&A within Learning Through (Chaoxing), and then we also have group work, group presentations... After class, it's the same, post-class exercises, and then uploading some of their more unique work after class..." |
| T9 | “我们在进行线上课的时候，我们会利用到的这些资源。再一个这些资源也是我们线下课的一个补充。有的时候我们留作业或者是考试，我们都会在线上进行。并且在线上我们会经常的和学生呢进行互动，或者是检验，或者是督促他们学习，通过留作业的形式通过网上的留言的形式，或者是上传自己作品的形式等多种形式。” | "When we conduct online classes, we utilize these resources. Furthermore, these resources are also a supplement to our offline classes. Sometimes when we assign homework or exams, we do it online. And online, we frequently interact with students, or assess, or urge them to study, through forms like assigning homework, leaving online messages, or uploading their own work, and other various forms." |
| T10 | “老师们在上课的时候...课前课中，我们这个点名，都会课后发布作业，都会用到咱们的平台。” | "When teachers are in class... pre-class, in-class, our roll call, post-class homework assignment, all use our platform." |
| T11 | “课前现在课前的预习复习...但在课中来讲，那肯定就是整个教学都是数字化教学呗。然后课后的话，有时候一些作业是要求同学用，就是拍成视频作业交过来的...” | "Pre-class, now pre-class preview and review... But during class, it certainly means the entire teaching is digital teaching. Then after class, sometimes some homework requires students to, for example, shoot a video and submit it..." |
| T13 | “课前...我每一个章节...我都可能会给他们发布这样一些章节的导读。那么导读里边就可能会包括一些课程思政的元素...那么在课后的话...发一些题目作为一个任务让他们去完成...也可能会让他们进行一些知识点框架的总结，或者说去发布一些思考题，甚至是干脆就组织一个活动...”或许部分教师在考量教学质量问题时，仍会回归传统课堂模式。我们称之为‘手机停车场’，他们会专门收走学生的手机，确保学生上课时无需携带。 | "Before class... for each chapter... I might issue them a chapter guide. The guide might include some elements of curriculum ideology and politics... Then after class... send out some questions as a task for them to complete... might also have them summarize the knowledge point framework, or post some thought questions, or even just organize an activity..."  "Perhaps some teachers, considering the issue of teaching quality... will still return to traditional classroom use. We call it ‘Phone parking lot’, and they specifically put away students’ phones so that they can go to class without phones." |
| T14 | “教学资源库主要是在智慧树上，然后是共享的，这个是所有的内容全部都共享。教师的授课课件，还有一些数字，还有一些增值服务，全部是实现共享的。” | "The teaching resource library is mainly on Wisdom Tree (ZhiHuiShu), and it is shared; all content is fully shared. Teachers' lecture courseware, and some digital resources, and some value-added services are all shared." |

**3.2.4.Application in Practical Teaching**

| **Participant** | **Original Chinese Quote** | **English Translation** |
| --- | --- | --- |
| T2 | “针对实训课，他可以去预约实训室。我们有一些相对比较简单的一些操作，学生在课前通过预习，然后通过预约实训室，基本上学生就以小组单位，我们要求他们提交视频，提交到课程平台上...”“像我们的综合护理实训或者在急救课程里面用的，急危重症课程里面用的比较多（高端模拟人）...”“虚拟穿刺，什么临床思维系统...插胃管，插导尿管一些这些基本的一些东西...”“我们像最后的话，我们的考试基本上是第一是线上成绩，线上的每一次...然后还有一个就是技能考核...”“我们在临床实习之前我们会有一个综合技能的这样的一个多站式的考核。我们在多站式的考核考完了以后，我们立马从后台里面就能导出所有的数据...”“我们大概投20个重点班，我们跟学校能实行同步交互...通过数字化的可视化中心...进行实时的这样的一个听课...” | "For practical training courses, they can book training rooms. For some relatively simple operations, students preview before class, then book the training room, basically students work in groups, we require them to submit videos, submit them to the course platform...""Used more in our comprehensive nursing training or in emergency courses, in critical care courses (high-end simulators)...""Virtual puncture, clinical thinking systems... inserting gastric tubes, inserting urinary catheters, some of these basic things...""As for final assessment, our exams basically consist of, first, online scores, every online... and then there is also a skill assessment...""Before the clinical practice week, we have a multi-station assessment of comprehensive skills. After the multi-station assessment is finished, we can immediately export all the data from the backend...""We have about 20 key partner classes (hospital-based classes), we can achieve synchronous interaction with the school... through the digital visualization center... conduct real-time class observation..." |
| T3 | “虚拟仿真实验室是开展一些高端课程的录制...智慧化实验室用在实验教学上面和远程医院互动上面，他们和医院远程互动也是用那个东西。” | "The virtual simulation lab is used to record some high-end courses... The smart lab is used for experimental teaching and remote hospital interaction; their remote interaction with hospitals also uses that." |
| T5 | “比如说像分娩妊娠分娩的虚实结合...心肺听诊系统...操作一些关键环节的微视频。这个对于学生课后练习操作的时候，老师不在现场，他们可以通过看这些视频点的话也可以指导他们的操作...”“利用分娩的虚实仿真系统...像这些鼻饲法导尿法，我们一个虚实的侵入性操作插管的一个模型，综合性的这种模型...” | "For example, the virtual-real combination for childbirth... cardiopulmonary auscultation system... micro-videos of key operational steps. These are useful for when students practice operations after class and the teacher is not present; they can watch these videos to guide their operation...""Using the virtual-real simulation system for childbirth... things like nasogastric tube feeding, urinary catheterization, we have a virtual-real model for invasive intubation procedures, a comprehensive model of this kind..." |
| T6 | “在实训室的话，我们也是虚实结合，就是有实际的操作，有虚拟仿真...” | "In the training room, we also combine virtual and real, meaning there is actual operation and virtual simulation..." |
| T7 | “实训课的话我们也可以放一些视频，让他们先看视频。还有这些VR，这些虚拟的教学，我们也会应用到...” | "For practical training classes, we can also show some videos, let them watch the videos first. And these VR, these virtual teaching methods, we also apply them..." |
| T8 | “虚实结合，我觉得效果是相对来说会比较好...学生在做的过程当中...”“虚拟仿真对我们专业课来说不是特别的用处不是特别大，但是对于基础课我觉得它还是非常好的。像我们解剖他在这一块他就做的很好...” | "The combination of virtual and real, I think the effect is relatively better... while students are doing it...""Virtual simulation isn't particularly useful for our professional courses, but for basic courses I think it's very good. Like our anatomy department does it very well in this area..." |
| T9 | “虚拟仿真实验室还在建设中。但是我们健康评估、急救等确实有虚拟的模拟人体模型，让学生进行操作和实践，提升一些实践操作能力。我们附属医院还有远程的这种模拟医疗系统，可以让学生通过视频的方式与临床一线的专家进行交流和探索...” | "The virtual simulation laboratory is still under construction. But we do have virtual simulation manikins for health assessment, emergency care, etc., allowing students to operate and practice, enhancing some practical operation skills. Our affiliated hospital also has a remote simulation medical system, allowing students to communicate and explore with frontline clinical experts via video..." |
| T11 | “在我们的实践课程中，许多一线教师认为使用模型比数字模拟更能取得良好效果，这种体验可模拟游戏化活动。但本人认为其效果不如使用模型。” | "In our practical courses, many frontline teachers believe that using models yields better results than digital simulation,the experience can resemble a game-like activity.I feel it’s not as effective as using a model." |
| T12 | “而这个虚拟仿真实训基地，一般我们都是用来进行实验课的。实验课其实我们在很多的专业当中都实现了虚拟仿真。比如说口腔，比如说老年，还有像我们说内科，医护等等。” | "And this virtual simulation training base is generally used by us for experimental classes. In fact, we have achieved virtual simulation in many majors for experimental classes. For example, stomatology, geriatrics, and like we said internal medicine, nursing, etc." |
| T14 | “虚拟仿真的这个我们的静脉虚拟静脉穿刺实训室...上课率也是也就是是百分之百的。也就是说你只要上静脉穿刺、静脉输液，那么一定是要先在虚拟仿真训练室进行练习，才去到这个实训室进行这个模型人的练习的。还有一个就是模拟医院...在学生实习之前，要到模拟医院进行四个学时的模你练习。”“学生的护理技能大赛...平时训练的过程中，我们也是去用一些虚拟仿真的系统，数字化的一些系统，包括数字化的一些考核资源去给学生来进行训练的。” | "The utilization rate of our virtual simulation venipuncture training room... is 100%. That means whenever you have venipuncture or intravenous infusion classes, you must first practice in the virtual simulation training room before going to the practical training room to practice on the manikins. Also, there is the simulated hospital... Before students go for internship, they need to undergo four credit hours of simulated practice in the simulated hospital.""For the students' nursing skills competition... during the usual training process, we also use some virtual simulation systems, digital systems, including digital assessment resources to train the students." |

**3.3.Systemic Tensions and Barriers**

**3.3.1. Policy Practice Gap**

| **Participant** | **Original Chinese Quote** | **English Translation** |
| --- | --- | --- |
| **T2** | “虽说学校有好多数字资源，但还是有部分老师和学生没把它们充分利用起来，这就造成了资源浪费。” | “Although the school possesses a wealth of digital resources, some teachers and students fail to fully utilize them, resulting in a waste of resources.” |
| **T5** | “另外第三个我觉得可能在制度上面的话也有一些问题...也就是说如果是说有一些很好的一些硬件，我们想要申购的话，有时候程序会比较麻烦。” | “Additionally, third, I think there might be some problems with the system... That is, if we want to purchase some good hardware, the procedures can sometimes be quite troublesome.” |
| **T6** | “还有一个就是学校配套的政策...比方说我建课花了了这么多的精力和体力...那我是不是奖励也没有...所以我觉得就配套机制的问题。” | “Another thing is the school's supporting policies... for example, I spent so much energy and physical strength to build the course... so I don’t get any reward either... so I think it’s the problem of the supporting mechanism.” |
| **T10** | “第一个因素就是学校的支持力度...但是学校明确说了，我不要报软件性的，只要硬件。也就是说他现在还没有把数字化的发展这一块，把它纳入到非常重要。” | “The first factor is the level of support from the school... But the school clearly stated, ‘Don’t propose software, only hardware.’ That means they have not yet prioritized digital development as very important.” |
| **T11** | “领导的支持肯定是最重要的...他只要有政策制度的支持，下面老师都愿意干的...领导大刀阔斧直接给我们砍成60，那就可能建不起来。” | “Leadership support is definitely the most important... As long as there is policy and institutional support from the top, teachers below are willing to do the work... The leader decisively cut it to 600,000, so it probably can’t be built.” |
| **T14** | “一个就是数字化的资源的质量问题...我们也去参考一些其他的资源，但是发现有一些资源来源太广泛，随意性也比较强。” | “One is the quality of digital resources... we also refer to other resources, but find that some resources have sources that are too broad and are somewhat arbitrary.” |

**3.3.2. Readiness Divide**

| **Participant** | **Original Chinese Quote** | **English Translation** |
| --- | --- | --- |
| **T2** | “年长教师对新平台的使用往往持抵触态度……而年轻教师则学习能力更强……我认为不同年龄段的教师都需要持续学习……” | “For older teachers, using new platforms feels very resistant ... whereas younger teachers learn quickly...I think teachers of different ages...we need to keep learning...” |
| **T4** | “学生呢在线上学习的时候，他的这个主动性、自觉性是不足的...可能跟这个个体差异要求比较高。” | “When students are learning online, their initiative and self-discipline are insufficient... probably requiring high individual differences.” |
| **T5** | “教师相关因素是重要影响因素 . ... 其中最关键的是教师自身的认知 . ... 他们尚未意识到我们已进入数字时代。” | “Teacher-related factors are a significant influencing factor. ... The most important one is the teacher’s own perception. ... He or she doesn’t realize we have entered a digital era.” |
| **T6** | “学生自控力不够，用数字平台的时候老容易走神，根本没法集中精力学习。” | “Students’ insufficient self-control leads to a tendency to be distracted when using digital platforms, making it difficult for them to focus on their studies.” |
| **T8** | “第二个的话，就是教师层面。我们现在不是每一个老师都能够很好的去运用这个数字化的教学...而且他会有的老师他会有一点抵触情绪...” | “Second is the teacher level. Not every teacher among us can use digital teaching well now... Moreover, some teachers even have a bit of resistance...” |
| **T11** | 在教学能力竞赛中，有些老师可能觉得采用直播教学、让学生实时弹幕互动等手段过于花哨，毫无实际价值。但年轻教师们显然认为这种形式效果显著，完全可以用于试讲环节。 | “When it comes to teaching ability competitions... some teachers may think that you use so much, such as live streaming and having classmates post bullet comments on site... They think it’s fancy and worthless. Young teachers will definitely think that this form is good and can be applied in trial classes” |
| **T13** | “学生们在这样一个时代所接触手机互联网特别多...他们就有可能会出现复制粘贴，可能就会出现这种甚至于作弊的方式。” | “Students in this era are exposed to mobile phones and the internet extensively... they might resort to copy-pasting, or even cheating.” |

**3.3.3. Technical Operational Barriers**

| **Participant** | **Original Chinese Quote** | **English Translation** |
| --- | --- | --- |
| **T1** | “第二个因素就是网络因素，就是硬件方面的因素...以前我们的校区就在旧的校区的时候，因为它的一个网络分部的这个基站不太多，所以就可能会比较差，特别到一楼的时候就会比较差...所以也会影响到我们在课中测试我们老师的一个开展。” | “The second factor is the network factor, the hardware aspect... Previously, when our campus was in the old location, because the distribution of network base stations wasn’t great, the signal could be poor, especially on the first floor... This also affected our in-class testing and the teachers’ implementation.” |
| **T5** | “第一个网络上面，肯定就是要通过这些建设，让整个学校的网络信息更顺畅一些...然后一体机等等这些硬件设施要跟上。” | “Regarding the first point about the network, we definitely need to improve the construction to make the school’s network information flow more smoothly... And hardware facilities like all-in-one computers need to be upgraded.” |
| **T6** | “平台都统一用的是这个超星是吧？...不联动...比方说我说考试系统，考完之后我登录的这一个我这个成绩最终我要导入到这一个什么教务系统里面去。” | “Are all platforms unified using Chaoxing?... They are not interconnected... For example, after the exam system finishes, the scores I log in ultimately need to be imported into the academic management system.” |
| **T7** | “有些软件确实不够友好，内部网络有时也不够便利……有时候我们很难登录……我希望它能变得更友好、更方便，越简单越好。” | “Some software is indeed not very user-friendly, and sometimes the internal network is not very convenient... Sometimes it is difficult for us to log in... I hope it becomes more user-friendly and convenient to use, the more stupid the better.” |
| **T11** | “有一些虚拟仿真...他要下新的软件，然后下新的APP，然后还要扫...输入什么码，输入完码之后还得这样那样那样就半天了，然后弄出来你也不知道啥样的...不好用。” | “Some virtual simulations... require downloading new software, new apps, then scanning... inputting some code, and after inputting the code, you have to do this and that, it takes forever, and then you don’t even know what comes out... Not user-friendly.” |

**3.3.4. Workload Pressures**

| **Participant** | **Original Chinese Quote** | **English Translation** |
| --- | --- | --- |
| **T1** | “可能对于学生来说，他们在利用手机的时间就可能会太长了...但是这样子学生的这个学习任务负担就非常，所以这个是一个原因就是我们自己在后面实施的时候也要协调一下，要平衡一下。” | “For students, the time they spend using their phones might become too long... but this way the students’ learning task burden becomes very heavy, so this is one reason... we need to coordinate and find a balance ourselves later.” |
| **T2** | “老师他的一个课前工作量的一个增加，课前课后工作量的增加。” | “It increases the teacher’s pre-class workload, pre-class and post-class workload.” |
| **T6** | “老师的时间不充裕。第二教师的兴趣不高，我觉得最重要的因素在于教师，就是太忙了...还有教师倦怠的问题。” | “Teachers don’t have enough time. Second, teachers’ interest isn’t high. I think the most important factor lies with the teachers—they are just too busy... There’s also the problem of teacher burnout.” |
| **T8** | “什么事情在刚开始的时候都是增加工作量的，目前为止是增加了的。但是如果说真的能够把这个东西...我觉得将来是肯定能够减少我们的工作量的。” | “Everything initially increases the workload, and so far, it has increased. But if we can really make this thing work... I think it will definitely reduce our workload in the future.” |
| **T9** | “现在线上课程比较多，学生对这个数字化教学的接受度和参与度还是参差不齐的。因为学生就觉得增加了他们的负担...” | “There are relatively many online courses now, and students’ acceptance and participation in digital teaching are still uneven. Because students feel it increases their burden...” |

**3.3.5 Professional Role Dissonance**

| **Participant** | **Original Chinese Quote** | **English Translation** |
| --- | --- | --- |
| **T1** | **“我们毕竟不是专业的技术人员，所以我们老师在进行数字化转型教学方面，一些技术的一个掌握，可能需要用到技术人员来帮助...但是很多学校可能是目前没有这帮人来帮助我们的专任老师去解决这些问题。”** | **“We are, after all, not professional technical staff. So when it comes to mastering some of the technologies for digital transformation teaching, our teachers may need the help of technical personnel... but many schools may not currently have such people to assist our full-time teachers in solving these problems.”** |
| **T2** | **“今年我也是个新手……因为我一直在管理学生技能竞赛。但说到数字教育技能，那完全是另一个领域，我需要进一步学习。”** | **“I am also a beginner this year... because I have been managing student skill competitions. But when it comes to digital education skills, it's a completely different field, and I need to learn more”** |
| **T5** | **“另外第三个我觉得可能在制度上面的话也有一些问题...也就是说如果是说有一些很好的一些硬件，我们想要申购的话，有时候程序会比较麻烦...老师们提出需求...但是因为这两年没做完之后，你会发现这两年大家各个教研室主任们的话，可能就比较不想申购。”** | **“Additionally, third, I think there might be some problems with the system... That is, if we want to purchase some good hardware, the procedures can sometimes be quite troublesome... Teachers put forward demands... but because in the past two years after not doing it, you’ll find that in these two years, the heads of various teaching and research offices may be less willing to apply for purchases.”** |
| **T8** | “然后第三个的影响因素就是学生他自己...就是他没有我们没有很好的方式去检测他是真他有没有真的在用，这是一个很大的问题。” | “And the third influencing factor is the students themselves... That is, we don’t have a good way to detect whether they are truly using it, whether they are really learning. This is a big problem.” |
| **T10** | “课程的建设的质量说实在话还不是特别好。老师们可能顶多就是放一些课件上去，然后自己比如说在电脑上自己录屏，一些讲课的一的视频放上去，就是在这个做的不是很精细。” | “To be honest, the quality of course construction is not particularly high. Teachers might at most upload some courseware, and then, for example, record their own screen on the computer, put up some videos of their lectures – it’s just not done very meticulously.” |
| **T14** | “一个就是数字化的资源的质量问题。我们虽然是我们自己也录制了一些在线课，但是弄完之后发现不太喜欢再去回头看。” | “One is the quality of digital resources. Although we have recorded some online courses ourselves, after finishing, we find we don’t really like to look back at them.” |

**3.4. Strategies for Educational Digital Transformation**

**3.4.1. Infrastructure Investment**

| **Participant** | **Original Chinese Quote** | **English Translation** |
| --- | --- | --- |
| T1 | “首先第一个很重要的一个影响因素就是经济条件，这是必定的对吧？对这个经济的一个投入，就是财政方面这个投入是直接就决定你这个成果规模的大与小。我们研究所建立了一个信息技术小组，主要任务是支持信息资源的开发和构建在线课程框架。” | "The first very important influencing factor is economic conditions, that's certain, right? The financial investment directly determines the scale of your outcomes, whether large or small.Our institute has established an information technology group whose main task is to support the development of information resources and the construction of online course frameworks." |
| T5 | “网络上面，肯定就是要通过这些建设，让整个学校的网络信息更顺畅一些。然后一体机等等这些硬件设施要跟上。” | "Regarding the network, it's necessary to make the school's network information flow more smoothly through these constructions. Then hardware facilities like all-in-one PCs need to keep up." |
| T7 | “那肯定是全方位的提升，这个软件和硬件的提升。还有就是...”“软件和硬件要跟得上。现在我们学校也开始做一些多媒体的教师这些智慧教师这些我们也在建设，这些都是不断的进步，实际上是有不断的投入的。” | "It definitely requires comprehensive improvement, improvement in both software and hardware. And also...""Software and hardware need to keep up. Now our school is also starting to do some multimedia classrooms, these smart classrooms we are also building; these are constant improvements, there is actually continuous investment." |
| T10 | “...第一个就刚才提到的资金的问题。那就是从咱们领导的重视程度上，学校层面上面对吧？应该加大资金的支持力度。” | "... The first is the funding issue just mentioned. That comes down to the attention from our leadership, at the school level, right? Should increase financial support." |
| T12 | “第一个就是资金的投入。我们在建设这个平台的时候，肯定需要一些资金的投入，像软件、设备等等。解决的办法，一个是希望学校能够给予支持，更多的投入。另一方面可以申请教育科研基金。再一个我们可以进行的校企合作，让这个企业来进行帮助。” | "The first is financial investment. When building this platform, we definitely need some financial investment, for things like software, equipment, etc. Solutions include hoping the school can provide support, more investment. On the other hand, we can apply for educational research funds. Also, we can engage in school-enterprise cooperation, letting enterprises provide help." |
| T14 | “第二个就是资金的投入，因为数字化确实是需要提供一些足够的资金支持的。你比如说基础设施建设，教育资源的采购，教师培训等等，都需要进行一个比较大的资金投入。” | "The second is financial investment, because digitalization indeed requires sufficient financial support. For example, infrastructure construction, procurement of educational resources, teacher training, etc., all require relatively large financial investment." |

**3.4.2. Educator Development**

| **Participant** | **Original Chinese Quote** | **English Translation** |
| --- | --- | --- |
| T1 | “第二个因素就是老师的信息化素养，老师的信息化素养它也会影响我们对于数字化教学的一个推行跟应用...我们学校会有一个信息中心。我们的信息中心也会定期向我们的在校老师进行一些新进展新技术的一个介绍和培训。也可以让我们了解更多一些新型的一些数字化资源的一些建设方法，这也可以提高我们的一个使用的一个兴趣。这还有的就是我们自己学院的话，还有的就是我们会做比我们建设了国经，建设了省经，还有获得了教学能力大赛的一些自己的一个团队进行这个经验的分享。也可以带动没有参与过这些项目的老师也能够参与进来。” | "The second factor is teachers' information literacy; teachers' information literacy also affects our promotion and application of digital teaching... Our school has an Information Center. Our Information Center also regularly provides introductions and training on new developments and technologies to our in-service teachers. This also lets us learn more about new methods for building digital resources, which can increase our interest in using them. Also, within our own college, we have teams that have built national-level quality courses, provincial-level quality courses, and won teaching competence competitions; they share their experiences. This can also motivate teachers who haven't participated in such projects to get involved." |
| T2 | “我们的信息中心定期为学校教师介绍新技术和新进展，并进行培训，以提高他们的数字技能” | "Our Information Center regularly conducts introductions and training sessions on new technologies and advancements for teachers in school, aiming to enhance their digital skills" |
| T5 | “教师因素上面的话，我觉得还是要多培训，多学习。要首先一个是要知道，现在有这么多的，现在是进入数字化的一个时代了，有而且有这么多这么好的一个数字的资源，还有手段可以使用，可以让你的教学的话更有趣，更生动一些。另外的话如果是说有一些好的手段跟教学方法等等，一定要多宣传。” | "Regarding teacher factors, I think more training and learning are still needed. First, they need to know that we are now in a digital era, and there are so many good digital resources and methods available that can make your teaching more interesting and vivid. Also, if there are some good methods and teaching approaches, etc., they must be promoted more." |
| T8 | “教师层面的话，这个就我觉得只能是多培训了吧。然后并且要制定一些督导的计划里面，像我们学校现在就是这样的督导计划里面是他的有没有运用数字化的教学，是放在他的评分里面的。” | "At the teacher level, I think the only way is more training. And then, also need to establish some supervision plans; like in our school now, in such supervision plans, whether they use digital teaching is included in their evaluation score." |
| T9 | “第二个就是需要加大师资队伍的建设，可能老师参差不齐，需要具备使用数字化教学工具和平台的一些技能。另外在教学过程中的一些教学策略、教学方法都应该有提升。是不是可以通过举办培训、研讨会、派老师出去学习，或者招聘有经验的老师，或者邀请专家进行指导，来提升整个师资队伍的数字化教学能力。” | "The second is the need to strengthen the teaching staff development; teachers' abilities are uneven, they need skills to use digital teaching tools and platforms. Also, teaching strategies and methods during the teaching process should be improved. Can we enhance the entire teaching staff's digital teaching capability by organizing training, seminars, sending teachers out for study, recruiting experienced teachers, or inviting experts for guidance?" |
| T13 | “目前大多数教师仍处于探索阶段，因此未来需要建立系统的培训机制，以提升其数字化教学能力” | "The majority of teachers are currently in the exploratory phase, necessitating the establishment of a systematic training regime in the future to enhance their digital teaching capabilities" |
| T14 | “第三个我觉得是教师培训，因为我前面做课题的时候，做过这个t-pack的，发现有很多老师他就是有这个心，但是在操作起来困难重重。就是因为他没有掌握一些数字化的一些技能和知识，所以要给教师提供一些相关的培训和支持，这样他才能够有效的运用数字化工具和资源进行教学。” | "The third I think is teacher training. Because when I did a project on TPACK before, I found many teachers have the intention, but face numerous difficulties in operation. Precisely because they haven't mastered some digital skills and knowledge, it's necessary to provide teachers with relevant training and support so they can effectively use digital tools and resources for teaching." |

**3.4.3. Resource Innovation**

| **Participant** | **Original Chinese Quote** | **English Translation** |
| --- | --- | --- |
| T1 | “老师获取应该是这么说，获取教学的信息化素材的渠道不够，获取信息化教学的取素材不够，就只能够依靠自己去建设，就自去创造的话，那你看时间、经济这条件也会受到限制。所以这种教学资源的获取的素材太少了。” | "To put it this way, teachers' channels for acquiring informational teaching materials are insufficient; there aren't enough sources for informational teaching materials. They can only rely on building them themselves. If they have to create them on their own, then you see, time and economic conditions become limiting factors. So, the sources for acquiring such teaching resources are too few." |
| T5 | “针对于数字资源，我就觉得可能不能包括学校政策层面的话，不能只是追求它的量，也要体现他的质。要怎么给他这些数字资源建设的更好，更有实用性，更有观赏性一些。” | "Regarding digital resources, I think perhaps, including at the school policy level, we cannot just pursue quantity, but also reflect their quality. How to make these digital resources better constructed, more practical, and more visually appealing." |
| T6 | “我觉得现在的大体育建设，但是实际上我觉得这种数字化，其实无论是建什么样的东西，我觉得最大目的应该是共建和共享。实际上现在共建的话，我牵头建，你又不愿意附属。所以现在就搞得你也建我也建。我现在觉得最大的烦恼就是不见了就没有共享，并没有实现，就是节约教育成本。” | "I think currently there is large-scale construction, but actually, I think this digitalization, regardless of what is built, the primary goal should be co-construction and sharing. Actually, now with co-construction, if I lead the construction, you are unwilling to be subsidiary. So now it ends up with you building and me building. My biggest frustration now is that without co-construction there is no sharing, and the goal of saving educational costs is not achieved." |
| T10 | “深度还不够，这个大家都在用了，但是这个深度就是没有更多的去创作一些自己原创性的一些数字化的一些资源。比较少，还是那个问题，这个资金的支持力度不够是吧？” | "The depth is still insufficient; everyone is using it, but the depth lies in not creating more of our own original digital resources. There are relatively few, it's still that problem, isn't it? The level of financial support is insufficient, right?" |
| T14 | “我们需要提升数字资源质量，鼓励原创内容创作，并通过平台促进资源共享” | "We need to enhance the quality of digital resources, encourage the development of original content, and facilitate resource sharing through platforms" |

**3.4.4 Incentive Mechanisms**

| **Participant** | **Original Chinese Quote** | **English Translation** |
| --- | --- | --- |
| T1 | “第一个方法当然就是以项目带动了，对吧？就是比如你国家精品课程，省级精品课程，包括教学能力大赛。那我们在学在这个鼓励机制上面，在这些项目它会有对应的鼓励机制。” | "The first method is, of course, to drive it through projects, right? For example, national-level quality courses, provincial-level quality courses, including teaching competence competitions. In terms of incentive mechanisms, these projects have corresponding incentives." |
| T2 | “学校将根据教师的数字化教学表现提供相应的政策支持和激励措施，从而提升他们的积极性。” | "The school will provide corresponding policy support and incentives based on the teachers’ digital teaching performance, thereby enhancing their enthusiasm" |
| T4 | “应建立科学的评估机制，涵盖学生评价、教师评价和课程评价，以持续提升数字化教学的质量。” | "A scientific assessment mechanism should be established, encompassing student evaluations, teacher evaluations, and course evaluations, to continuously enhance the quality of digital teaching" |
| T6 | “还有一个就是学校配套的政策。比方说我建错了这么多的精力和体力，那我是不是有奖励也没有。所以我觉得就配套机制的问题。” | "Another is the supporting policies from the school. For example, if I put in so much effort and energy into building, is there any reward? No. So I think it's a problem of the supporting mechanism." |
| T8 | “并且要制定一些督导的计划里面，像我们学校现在就是这样的督导计划里面是他的有没有运用数字化的教学，是放在他的评分里面的。所以这样的话也是鼓励老师的一个方法。” | "And need to establish some supervision plans; like in our school now, in such supervision plans, whether they use digital teaching is included in their evaluation score. So this is also a way to encourage teachers." |
| T10 | “老师的话一个就是扭转观念，应该让学校这块政策方面跟我们的绩效，跟我们的一个奖励机制挂钩，这样的话可能会调动老师们的一个积极性。” | "For teachers, one aspect is to change their mindset; the school's policy in this area should be linked to our performance and our reward mechanism. This might motivate teachers." |
| T13 | “学校应建立相对有效的教学绩效考核体系，对认真推行数字化教学方法的教师给予相应的系数奖励。肯定一个老师如果是像以前那样简单的上传统课堂，跟一个老师去上信息化数字化的课堂，他们两个体现出的工作量是完全不一样的。其实我们学校现在有一个系数的问题，比如说我按照很普通的方式去上一节课就算一个学时。但是如果有一个老师他成功申报了，比如像是混合式教学，线上线下结合式教学这种方法的话，他可能从一个学时上就给他0.1的系数来体现他的工作量。其实我认为这方面我们学校还是做的挺好的，这样一个方式也可以促进老师们去更好的来开展这样一个教学化资源。” | "Schools should have a relatively effective teaching performance system, which grants corresponding coefficient rewards to teachers who diligently implement digital teaching methods.Certainly, if a teacher simply teaches traditional classes like before, compared to a teacher who teaches informationalized, digital classes, the workload they reflect is completely different. Actually, our school now has a coefficient system. For example, if I teach a class in a very ordinary way, it counts as one credit hour. But if a teacher successfully applies for, say, blended teaching, online-offline combined teaching methods, they might get a 0.1 coefficient added per credit hour to reflect their workload. Actually, I think our school does quite well in this aspect; such a method can also promote teachers to better develop such informational teaching resources." |

**3.4.5. Industry-Education Integration**

| **Participant** | **Original Chinese Quote** | **English Translation** |
| --- | --- | --- |
| T1 | “如何把我们的已经做好的数字化资源，让他能够得到更大的一个社会影响力。比如有所谓的院企合作、校企合作，进行把我们的数字化教学资源也可以推开到临床的教学里面。这样子是会让我们的数字化建设的资源的利使用率能够得到更好的一个提升，或者是让他们的影响面会得到扩展的更大，也可以提升他们临床教学的一个效率还有质量。这是目前的一个问题，还是比较少这方面的一个校企合作，再从数字化转型这个切入点来说是比较少...我们正寻求与企业合作开发一款可在手机上运行的虚拟教学系统，提升学生的学习效果” | "How can we make our already developed digital resources gain greater social influence? For example, through so-called college-enterprise cooperation, school-enterprise cooperation, to extend our digital teaching resources into clinical teaching. This would allow for better improvement in the utilization rate of our digitally constructed resources, or expand their impact more widely, and also enhance the efficiency and quality of their clinical teaching. This is a current problem; there is still relatively little school-enterprise cooperation in this aspect, especially from the perspective of digital transformation as an entry point, it is relatively scarce..We are seeking collaboration with enterprises to develop a virtual teaching system that can be implemented on mobile phones, aiming to enhance students’ learning outcomes." |
| T3 | “从教学能力大赛、技能比赛和产业融合等方面，可以深入的一个人数字化赋能的一个。” | "From aspects like teaching competence competitions, skills competitions, and industry integration, we can deeply pursue digital empowerment." |
| T9 | “数字化转型可与远程医疗及教学相结合，使学生能与一线临床专家进行视频交流...学校是不是能够筹备一些资金，申请一些项目，或者可以去企业合作的方式来获得这种额外的资金的支持。” | "Digital transformation can be integrated with telemedicine and teaching, enabling students to engage in video communications with frontline clinical experts...Can the school raise some funds, apply for some projects, or adopt school-enterprise cooperation to obtain this additional financial support?" |
| T12 | “再一个我们可以进行的校企合作，让这个企业来进行帮助。” | "Also, we can engage in school-enterprise cooperation, letting enterprises provide help." |
| T14 | “这个数字化转型的核心，它其实对于护理来说最主要的目的就是更好的能够服务社会服务人民群众。所以在进行人才培养时要以我们的临床护理技能为导向，掌握目前的医疗卫生的需求，开发一些市场化需求导向的这种人才培养模式。比如说我们要和医院行业紧密联系，开展一些专题研究课题研究，形成一定的育人模式，带动学生接触最新技术和产业的发展动态。” | "The core of this digital transformation is actually the main purpose for nursing to better serve society and the people. So when cultivating talents, we should be guided by our clinical nursing skills, grasp the current needs of medical and health care, and develop market-oriented talent training models. For example, we need to closely connect with the hospital industry, conduct some specialized research projects, form a certain education model, and drive students to be exposed to the latest technology and industry development trends." |
